# Molecular and Morphological Toxicity of Diatom-Derived Hydroxyacid Mixtures to Sea Urchin *Paracentrotus lividus* Embryos

**DOI:** 10.3390/md17030144

**Published:** 2019-03-01

**Authors:** Luisa Albarano, Nadia Ruocco, Adrianna Ianora, Giovanni Libralato, Loredana Manfra, Maria Costantini

**Affiliations:** 1 Department of Marine Biotechnology, Stazione Zoologica Anton Dohrn, Villa Comunale, 80121 Napoli, Italy; luisa.albarano@szn.it (L.A.); nadia.ruocco@szn.it (N.R.); ianora@szn.it (A.I.); giovanni.libralato@unina.it (G.L.); 2 Department of Biology, University of Naples Federico II, Complesso Universitario di Monte Sant’Angelo, Via Cinthia, 80126 Napoli, Italy; 3 Institute for Environmental Protection and Research (ISPRA), 00144 Rome, Italy; loredana.manfra@isprambiente.it; 4 Department of Biology and Evolution of Marine Organisms, Stazione Zoologica Anton Dohrn, Villa Comunale, 80121 Napoli, Italy

**Keywords:** diatoms, genes, hydroxyacids, sea urchin

## Abstract

Oxylipins such as polyunsaturated aldehydes (PUAs) and hydroxyacids (HEPEs) are signaling molecules derived from the oxidation of polyunsaturated fatty acids. They are common in diatoms that constitute a major group of microalgae in freshwater and oceanic ecosystems. Although HEPEs represent the most common oxylipins produced by diatoms, little information is available on their effects on marine invertebrates, and most of the information has been obtained by testing individual HEPEs. Our previous studies reported that four hydroxyacids, i.e., 5-, 9-, 11-, and 15-HEPE, were able to induce malformations and a marked developmental delay in sea urchin *Paracentrotus lividus* embryos, which had not been reported for other oxylipins. Here, we tested a mixture of 5-, 9-, 11-, and 15-HEPE at different concentrations for the first time. The results showed that mixtures of HEPEs have synergistic effects that are much more severe compared to those of individual HEPEs: The HEPE mixtures induced malformations in sea urchin embryos at lower concentrations. Increasing HEPE mixture concentrations induced a marked increase in the number of delayed embryos, until all embryos were delayed at the highest concentration tested. At the molecular level, the HEPE mixtures induced variations in the expression of 50 genes involved in different functional processes, mainly down-regulating these genes at the earliest stages of embryonic development. These findings are ecologically significant, considering that during diatom blooms, sea urchins could accumulate HEPEs in concentrations comparable to those tested in the present study.

## 1. Introduction

Oxylipins are oxygenated fatty acids derivatives distributed in a wide range of organisms, including plants [1] and algae [2], where they are involved in the regulation of several physiological processes [3,4]. In diatoms, these molecules are produced through an enzymatic cascade activated after cell breakage [5,6,7]. In this process, specific lipoxygenases mediate the oxygenation of polyunsaturated fatty acids (PUFAs), mainly C16, C20, and C22 [8,9,10], producing fatty acid hydroperoxides (FAHs), which are direct precursors of a wide array of both poly-unsaturated aldehydes (PUAs) and fatty acid derivatives with hydroxy-, keto-, oxo-, and epoxy functionalities [7,11,12]. Following the demonstration by Miralto et al. [13] that these diatom secondary metabolites induced reproductive failure in copepods, many other studies have identified these negative effects on a wide variety of grazers [14,15]. Several studies have investigated how oxylipins affect embryo development in the sea urchin *Paracentrotus lividus*, which is a good model organism for eco-toxicological assessments [16,17,18]. Bioassays on sea urchin embryos have shown that two classes of oxylipins, i.e., PUAs and HEPEs, induce abnormalities and/or delay in sea urchin embryos [19,20]. Three abundant PUAs—decadienal, heptadienal, and octadienal—have been demonstrated to impair sea urchin embryo development in a dose-dependent manner, with decadienal showing the strongest effect (i.e., concentrations ranging from 0.5 to 2.5 μM for decadienal, from 1.0 to 6.0 μM for heptadienal, and from 2.0 to9.0 μM for octadienal) [19]. Moreover, individual tests with two highly distributed HEPEs (5- and 15-HEPE), revealed that these compounds were less active than PUAs [20]. In fact, these compounds induced an increase in the number of abnormal plutei in a higher range of concentrations (6–15 μM) than those used for PUAs. Although the toxicity of HEPEs was less severe compared to PUAs, these authors observed a strong delay in development, with embryos blocked at the early pluteus stage at 30 μM after 48 h post fertilization (hpf) [20]. When tested at higher concentrations (up to 100 μM), HEPEs induced an even stronger developmental delay, with all embryos blocked at the gastrula stage [21].

In the present study, we tested 5-, 9-, 11- and 15-HEPE mixtures on the sea urchin *P. lividus* to investigate how combinations of these compounds affected embryonic development. Previous studies have already shown a synergistic behavior of other oxylipins such as PUAs which induced stronger effects on the development of *P. lividus* compared to single PUAs [22]. Sea urchin eggs were fertilized, incubated with mixtures of four HEPEs (already tested separately [20,21]), and embryonic development was then followed until the pluteus stage at 48 hpf. In order to identify possible gene targets affected by HEPEs mixtures, we followed the variation of the expression levels of 50 genes involved in different functional processes during this developmental period [19,23,24].

## 2. Results

### 2.1. Effects of 5-, 9-, 11-, and 15-HEPE Mixtures on Sea Urchin Development

**Figure 1:**
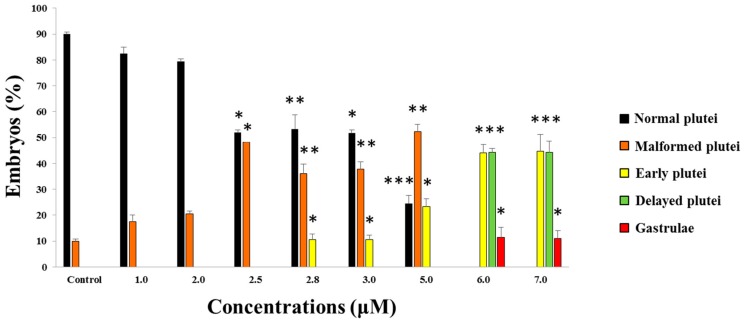
Percentage of normal, malformed, early, and delayed plutei and gastrulae in control (embryos grown in the absence of hydroxyacid mixtures) and treated samples with 5-, 9-, 11-, and 15-HEPE mixtures at the concentrations of 1.0, 2.0, 2.5, 2.8, 3.0, 5.0, 6.0, and 7.0 μM. Student’s t-tests (* *p* < 0.01, ** *p* < 0.001, *** *p* < 0.0001).

To determine the effects of 5-, 9-, 11-, and 15-HEPE mixtures on sea urchin embryo development, we used 6.0 and 7.0 µM as starting concentrations, since these were the concentrations that induced about 15% and 21.5% of malformed embryos and 6.1% and 12.4% of delayed embryos, respectively, when 5- and 15-HEPE were tested individually [20] (Figure 1).

**Figure 2:**
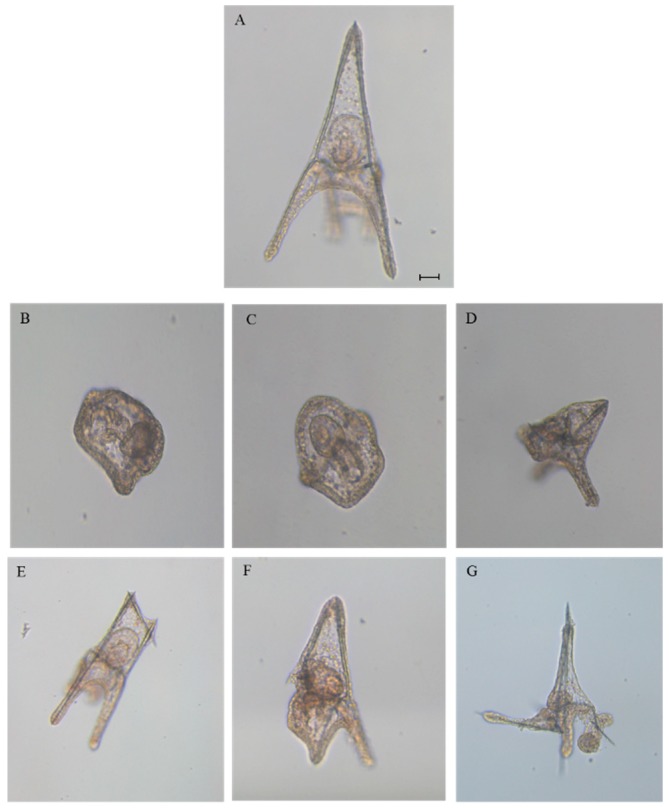
Photos (taken with Zeiss Axiovert 135TV microscope, 10× magnification, 0.30 numerical aperture) of (**A**) controls at the pluteus stage (at 48 h post fertilization (hpf); embryos in sea water without HEPE mixtures), (**B**) malformed gastrula, (**C**) early plutei, (**D**–**G**) malformed plutei after incubation with 5-, 9-, 11-, and 15-HEPE mixtures. Scale bar: 50 µm.

After adding the four HEPEs at these concentrations, we followed the development of *P. lividus* embryos, checking the following endpoints: (1) first cleavage division, leading to two blastomeres, at about 1 hpf, (2) pluteus stage at 48 hpf. After treatment with both HEPE mixtures, we observed 100% first mitotic division, but after 48 hpf, all embryos were delayed (Figure 1). Approximately 11% of embryos were malformed and still at the gastrula stage (Figure 2B; *p* < 0.05), 45% were apparently normal but at the early pluteus stage (Figure 2C; *p* < 0.001), and 44% of embryos resembled control pluteus embryos (Figure 2A), with only a slight reduction in body length (Figure 2D; *p* < 0.001).

**Figure 3:**
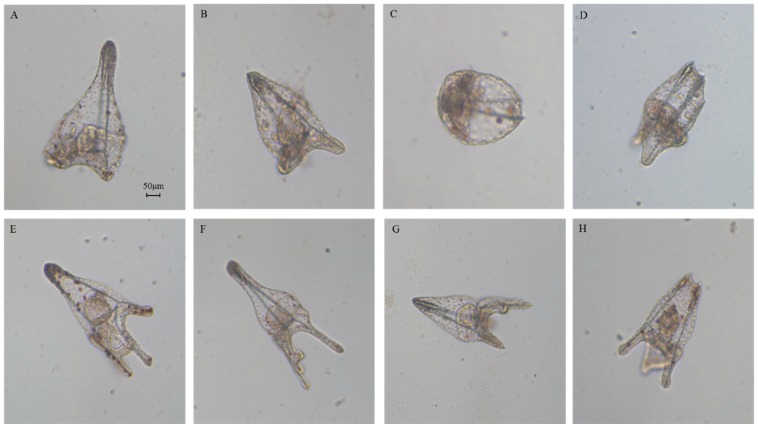
Photos (taken with Zeiss Axiovert 135TV microscope, 10× magnification, 0.30 numerical aperture) of (**A**) control (embryos in sea water without HEPE mixtures) at one week after fertilization, (**B**–**H**) abnormal embryos after incubation with 5-, 9-, 11-, and 15-HEPE mixtures at the concentration of 6.0 and 7.0 μM. Scale bar: 50 µm.

We followed embryonic development for one week post-fertilization to observe the fate of all the delayed embryos. Some embryos had developed beyond the pluteus stage but were malformed as seen in Figure 3B–D, not having the characteristic ampoule-like shape as in the control (Figure 3A [20]). Other embryos reached the pluteus stage even if they were malformed, with degraded arms and apex as seen in Figure 3E–H.

In the second experiment, the concentration of the four HEPEs was lowered to 5.0 μM. At this concentration, we observed 100% first mitotic division, and the delay in development was weaker. In fact, after 48 hpf, we observed 52% malformed plutei (Figure 2E–G; *p* < 0.01) and 23% still at the early pluteus stage as seen in Figure 2C; *p* < 0.05. At this concentration, no embryos were still at the gastrula stage.

We then further decreased the HEPEs concentrations in the mixtures to 3.0 and 2.8 µM; both treatments induced comparable results. In fact, at 48 hpf, we observed about 40% malformed plutei that were not delayed (as in the case of embryos reported in Figure 2E–G; *p* < 0.01); 10% of embryos were delayed and were still at the early pluteus stage (*p* < 0.05), and 50% were normal plutei.

Further decreasing the concentration of the HEPE mixtures to 2.5, 2.0, and 1.0 µM led to 100% first division. At 2.5 µM, no delayed embryos were observed, but almost 48% were malformed (*p* < 0.05). At 2.0 and 1.0 µM, no differences were observed with respect to the control embryos.

### 2.2. Gene Response to HEPE Mixtures

**Figure 4:**
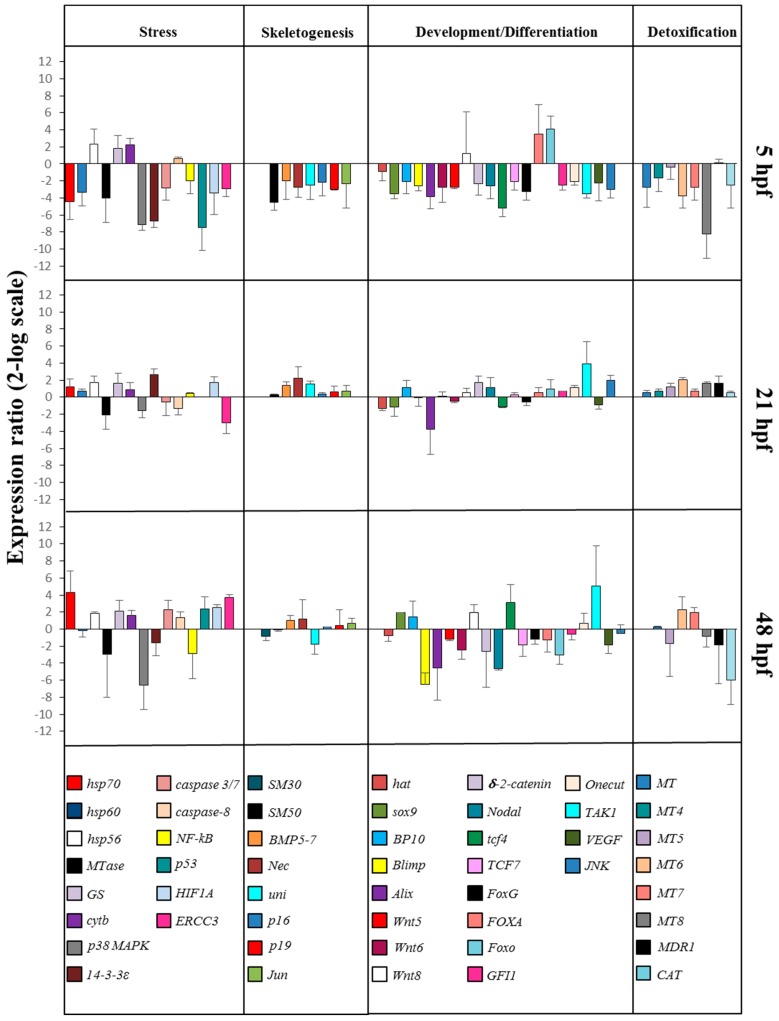
Real-time qPCR at blastula (5 hpf), gastrula (21 hpf), and pluteus (48 hpf) stages. Histograms show the differences in the expression levels of 50 genes involved in different functional processes: stress, skeletogenesis, development/differentiation, and detoxification. *Paracentrotus lividus* embryos were grown in the presence of 5-, 9-, 11-, and 15-HEPE mixtures at the concentration of 2.8 μM. Fold differences greater than ±2 (see red dotted horizontal guidelines at values of +2 and −2) were considered significant (see Supplementary Table S1 for the values).

On the basis of our morphological results, we incubated embryos with 5-, 9-, 11-, and 15-HEPE mixture at 2.8 µM to check the molecular response of *P. lividus* embryos to this treatment. This concentration was chosen because the delay and malformation effects were too strong at higher concentrations while they were lost or not apparent at lower concentrations. In order to study these effects at the molecular level, we followed the expression levels of 50 genes involved in different physiological processes and previously studied in response to individual HEPEs by real-time qPCR as seen in Figure 4 and Supplementary Table S1.

#### 2.2.1. Stress Genes

At the blastula stage, 10 genes were down-regulated with respect to the control: *hsp70*, *hsp60*, *Mtase*, *p38 MAPK*, *14-3-3ε*, *caspase 3/7*, *NF-kB*, *p53*, *HIF1A*, and *ERCC3*. Only *hsp56* and *cytb* were up-regulated, with respect to the control. At the gastrula stage three genes were targeted: *MTase* and *ERCC3* were down-regulated, whereas *14-3-3ε* was up-regulated with respect to the control. At the pluteus stage nine genes were targeted: *Mtase*, *p38 MAPK*, and *NF-kB* were down-regulated, whereas *hsp70*, *GS*, *caspase 3/7*, *p53*, *HIF1A*, and *ERCC3* were up-regulated with respect to the control.

#### 2.2.2. Skeletogenic Genes

At the blastula stage seven genes were down-regulated by the HEPE mixture: *SM50*, *BMP5/7*, *Nec*, *uni*, *p16*, *p19*, and *Jun*. At the gastrula stage (21 hpf) only *Nec* was up-regulated, and at the pluteus stage, (48 hpf) no genes were targeted.

#### 2.2.3. Genes Involved in Development/Differentiation

Gene expression analysis of genes involved in development/differentiation showed that, at the blastula stage, the genes *sox9*, *BP10*, *Blimp*, *Alix*, *WNT5*, *WNT6*, *δ-2-catenin*, *Nodal*, *tcf4*, *tcf7*, *FoxG*, *GFI1*, *Onecut*, *TAK1*, *VEGF*, and *JNK* were down-regulated and the genes *FoxA* and *FoxO* were up-regulated. At the gastrula stage, the gene *Alix* was down-regulated, and the genes *TAK1* and *JNK* were up-regulated. At the pluteus stage, seven genes were targeted: *Blimp*, *Alix*, *WNT6*, *WNT8*, *δ-2-catenin*, *Nodal*, and *Foxo* were down-regulated, and *tcf4* and *TAK1* were up-regulated.

#### 2.2.4. Genes Involved in Detoxification

At the blastula stage *MT*, *MT6*, *MT7*, *MT8*, and *CAT* were down-regulated. At the gastrula stage, only *MT6* gene was up-regulated. At the pluteus stage, *MT5* and *MT6* were up-regulated, whereas *MDR1* and *CAT* were down-regulated.

**Figure 5:**
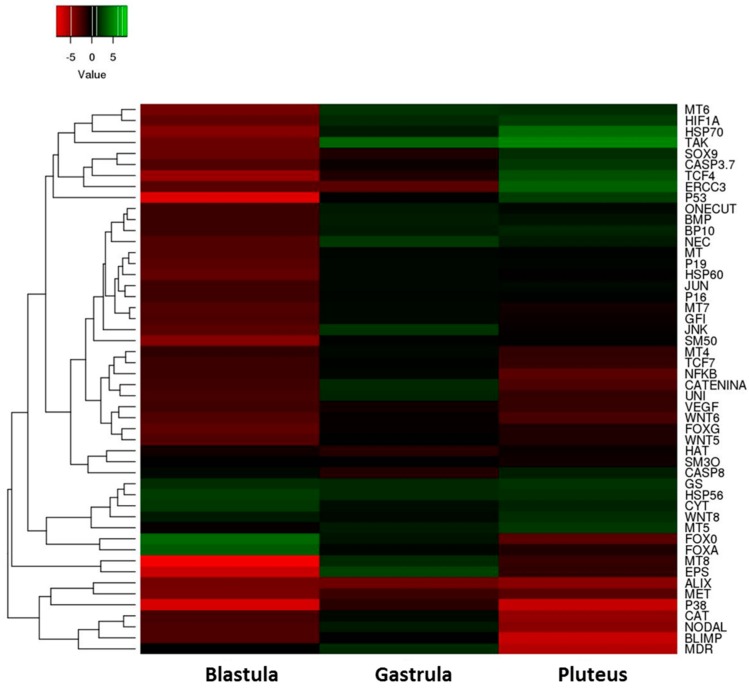
Heatmap (Heatmapper, available at the site www.heatmappear.ca) of differentially expressed genes in the three developmental stages (blastula, gastrula, and pluteus) after treatment with a 5-, 9-, 11-, and 15-HEPE mixture at the concentration of 2.8 μM. Color codes: red, negative values of gene expression (down-regulated genes with respect to the control, embryos developed in sea water without HEPE mixture); green, positive values of gene expression (up-regulated genes with respect to the control); black, genes for which there was no variation of expression with respect to the control.

These results are summarized in the heatmap reported in Figure 5 which shows differentially expressed genes in the three stages of sea urchin embryonic development.

We observed: (1) a high level of gene expression variability among the three different developmental stages; (2) almost all the genes analyzed showed a down-regulation of their expression after treatment with the mixture, especially at the blastula stage.

## 3. Discussion

Our work explored the noxious effects of diatom oxylipin mixtures, to which marine organisms are commonly exposed in their natural habitats. A previous study revealed that mixtures of the three PUAs decadienal, heptadienal, and octadienal acted in a synergistic way on sea urchin *P. lividus* embryonic development [22]. When tested separately, these compounds induced about 35% abnormal plutei at concentrations of 1.6 μM for decadienal, 3.0 μM for heptadienal, and 4.5 μM for octadienal [19]. Interestingly, when these PUAs were mixed, the same percentage of malformed plutei was observed at concentrations that were one-third of those used in individual tests [22].

To date, few studies have been conducted testing the effects of single HEPEs. Ianora et al. [25] tested the effects of a monoalgal diet with a 15S-HEPE-producing diatom, on the larval development of the copepod *Temora stylifera*. After 15 days of feeding, egg production and hatching success were significantly reduced, and most hatched nauplii appeared malformed with evident apoptotic regions in correspondence to the morphological abnormalities. However, most studies have tested the effects of pure HEPEs rather than those of diets rich in these compounds. Varrella et al. [20] reported that when sea urchin *P. lividus* embryos were exposed to 5- and 15-HEPE, these compounds induced a weaker toxicity when compared to PUAs in terms of number of malformed embryos. In fact, to induce the production of about 35% malformed plutei, higher concentrations (7 μM) of HEPEs were used in comparison to PUAs treatments (1.6 μM decadienal, 3.0 μM heptadienal, and 4.5 μM octadienal) [19,20]. Furthermore, single PUAs were able to induce apoptosis, whereas single HEPEs induced only a strong delay in *P. lividus* embryonic development even at the very high concentration of 100 μM [21].

We demonstrated for the first time that mixtures of 5-, 9-, 11-, and 15-HEPE can have very strong effects on sea urchin development compared to the single compounds. At high concentrations (6 and 7 μM) single HEPEs induced about 20–30% of delayed and malformed embryos [20]. At the same concentrations, HEPE mixtures induced 100% malformed and delayed embryos. Reducing the concentrations to 2.5 μM increased the number of malformed plutei (from 35 to 50%) and decreased the level of embryonic development delay. At even lower concentrations (2.0 and 1.0 μM), the effects of HEPE mixtures were not detectable, with no statistical differences with respect to the control samples.

All together these findings revealed that HEPEs in mixtures had a synergistic action, with increased harmful effects when tested together, as previously found for PUAs [22]. Moreover, the experiments with HEPE mixtures confirmed that HEPEs are able to induce a delay in the embryonic development of the sea urchin *P. lividus*, which has not been previously reported for other oxylipins.

Furthermore, our previous analysis with individual HEPEs demonstrated that 5- and 15-HEPE had few common molecular targets, specifically affecting different classes of genes at different development times. In particular, 15-HEPE switched on fewer genes than 5-HEPE, up-regulating mainly stress-related genes at a later pluteus stage. Also, 5-HEPE was stronger than 15-HEPE, targeting 24 genes, mainly at the earliest stages of embryo development, at the blastula and swimming blastula stages. The analysis of the variation of gene expression in embryos treated with the HEPE mixture agreed with our morphological results. Almost all genes involved in skeletogenesis (with the only exception of *SM30* and *BMP5/7*) and in development and differentiation processes (with the only exception of *hat* gene) were switched on, which may correlate with the presence of malformations and/or the strong delay observed at 48 hpf after treatment. Interestingly, we also found a different molecular response to HEPE and PUA mixtures. The molecular response to PUA mixture occurred later (48 hpf) [22] compared to HEPEs (5 hpf), but most analyzed genes were the same and were strongly down-regulated (Supplementary Figure S1). This suggests that with the HEPE mixture, the defensome [19] was compromised earlier, before reaching the gastrula and pluteus stages, which may explain why these sea urchin embryos could recover after treatment with PUAs [19], but not with HEPEs [20]. The defensome, as defined first for the sea urchin *Strongylocentrotus purpuratus* [26] and then for *P. lividus* [19], consists of integrated gene networks that allow an organism to defend itself against toxicants.

Overall, our investigation provides further evidence that diatom oxylipins can act as chemical defense molecules affecting the reproduction of grazers. Moreover, when tested in mixtures, they induced a synergistic effect, suggesting that their impact could be probably higher than those reported in previous studies testing individual compounds [19,20,21,27]. This is quite alarming, since marine organisms are exposed to several oxylipins at the same time. Very few data reported the natural ratios and abundance of HEPEs in diatoms. Cutignano et al. [11] reported the concentration of 0.22 pg/cell of 8-HEPE during the spring bloom in the Adriatic Sea in 2005. We calculated the daily ingestion rates of individual HEPEs as 0.022 μg [19]. Therefore, sea urchins would need to ingest this concentration for 30 days in order to accumulate 0.7 μg/mL, corresponding to 2.1 μM (concentrations comparable with those used in our mixture experiments in the present work).

Since sea urchins feed on benthic diatoms (the most important structural elements of the periphyton of seagrasses such as *Posidonia oceanica*, a natural food for sea urchins), which produce oxylipins [28], these results are of a significant ecological relevance and stimulate further testing of PUA and HEPE mixtures to better explore how their interactions may affect the reproductive success of marine invertebrates.

## 4. Materials and Methods

### 4.1. HEPE Mixtures and Morphological Analysis

The experimental procedures for gamete collection and incubation of fertilized eggs with HEPEs were according to Varrella et al. [20]. The four HEPEs were not added individually, but in mixtures after fertilization. The four HEPEs tested were the following:

- 5-hydroxy-6E,8Z,11Z,14Z,17Z-eicosapentaenoic acid (5-HEPE; Cayman Chemical, Ann Arbor, Michigan; purity ≥98%);

- 9-hydroxy-5Z,7E,11Z,14Z,17Z-eicosapentaenoic acid (9-HEPE; Cayman Chemical, Ann Arbor, Michigan; purity ≥98%);

- 11-hydroxy-5Z,8Z,12E,14Z,17Z-eicosapentaenoic acid (11-HEPE; Cayman Chemical, Ann Arbor, Michigan; purity ≥98%);

- 15-hydroxy-5Z,8Z,11Z,13E,17Z-eicosapentaenoic acid (15-HEPE; Cayman Chemical, Ann Arbor, Michigan; purity ≥98%).

Experiments were conducted in triplicates for different concentrations tested of HEPE mixtures (1.0, 2.0, 2.5, 2.8, 3.0, 5.0, 6.0, and 7.0 μM for each of the four HEPEs tested), using eggs from 10 different females. Control experiments consisted in fertilizing eggs in filtered sea water without HEPE mixtures. Embryonic development in the presence of HEPE mixtures was followed until 48 hpf, corresponding to the pluteus stage. Embryos were then fixed with formaldehyde and observed under a light microscope (Zeiss Axiovert 135TV, Carl Zeiss, Jena, Germany) to detect the percentage of abnormal plutei. Statistical analysis was performed using GraphPad Prism version 4.00 for Windows (GraphPad Software, San Diego, CA, USA).

### 4.2. Gene Expression by Real-Time qPCR

Collection of embryos at 5 (early blastula), 21 (early gastrula), and 48 (pluteus) hpf and total RNA extraction were performed using Aurum™ Total RNA Mini Kit (Bio-Rad), according to Ruocco et al. [29]. For further details on quantification and qualitative analysis of RNA, see Ruocco et al. [29]. For each sample, 1 µg of total RNA was retrotranscribed with an iScript™ cDNA Synthesis kit (Bio-Rad, Milan, Italy), following the manufacturer’s instructions.

The expression levels of 50 genes, previously analyzed in response to individual HEPEs, were followed by real-time qPCR [19,21,24,25], together with *Pl-Z12-1* [30] used as a control gene to internally normalize the data using REST software (Relative Expression Software Tool, Weihenstephan, Germany; version 1.9.12) [31,32]. Undiluted cDNA was used as a template in a reaction containing a final concentration of 0.3 mM for each primer and 1× FastStart SYBR Green master mix (total volume of 10 μL) (Applied Biosystems, Monza, Italy). PCR amplifications were performed in a ViiATM7 real-time PCR System (Applied Biosystems, Monza, Italy) thermal cycler using the following thermal profile: 95 °C for 10 min, one cycle for cDNA denaturation; 95 °C for 15 s and 60 °C for 1 min, 40 cycles for amplification; 72 °C for 5 min, one cycle for final elongation; one cycle for melting curve analysis (from 60 °C to 95 °C) to verify the presence of a single product. Each assay included a no-template control for each primer pair. To capture intra-assay variability, all real-time qPCR reactions were carried out in triplicate. Fluorescence was measured using ViiATM7 software (Applied Biosystems, Monza, Italy). The expression of each gene was analyzed and normalized against *Z12-1* gene, using REST software (Relative Expression Software Tool, Weihenstephan, Germany, version 1.9.12) on the basis of the Pfaffl method [31,32]. Relative expression ratios greater than ± 2 were considered significant. A non-parametric Kruskal–Wallis test was applied, followed by Dunn’s post-hoc test.

## Supplementary Materials

The following are available online at https://www.mdpi.com/1660-3397/17/3/144/s1; Figure S1: Synopsis of the patterns of up- and downregulation of different classes of genes in the sea urchin *P. lividus* in the presence of a 5-, 9-, 11-, and 15-HEPE mixture at 2.8 μM. Table S1: Data of expression levels are reported as a fold difference from control at blastula (5 hpf), gastrula (21 hpf), and pluteus (48 hpf) stages after treatment with the 5- 9-, 11-, and 15-HEPE mixture at 2.8 μM.
