# Supplementary material for: Molecular and Morphological Toxicity of Diatom-Derived Hydroxyacid Mixtures to Sea Urchin Paracentrotus lividus Embryos
Source: Mar Drugs. 2019 Mar 1;17(3):144. doi: 10.3390/md17030144 (PMC6470663; doi:10.3390/md17030144)
Supplement: Supplementary file 1 [file marinedrugs-17-00144-s001.pdf]

Supplementary Materials

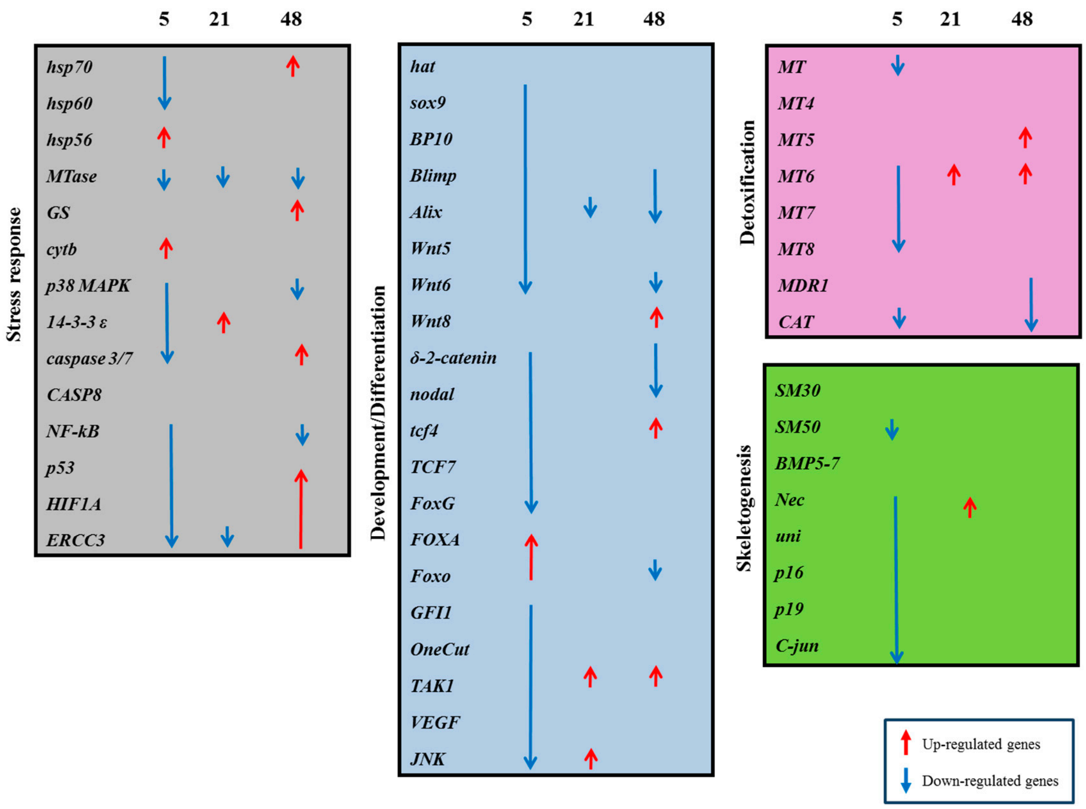

Figure S1. Synopsis of the patterns of up- and downregulation of different classes of genes in the sea urchin *P. lividus* in the presence of 5-, 9-, 11- and 15-HEPE mixture at 2.8 μM.

Table S1. Data of expression levels were reported as a fold difference from control at blastula (5 hpf), gastrula (21 hpf) and pluteus (48 hpf) after treatment with 5- 9-, 11- and 15-HEPE mixture at 2.8 μM. Fold differences greater than ± 2 were considered significant.

	**Blastula**	**Gastrula**	**Pluteus**
Stress Response			
hsp70	−4.4	1.2	4.3
hsp60	−3.3	0.7	−0.2
hsp56	2.4	1.7	1.9
Mtase	−4.0	−2.1	−3.0
GS	1.8	1.7	2.1
cytb	2.3	0.9	1.6
p38 MAPK	−7.1	−1.6	−6.5
14-3-3Ɛ	−6.7	2.6	−1.6
caspase 3/7	−2.8	−0.6	2.3
caspase-8	0.6	−1.3	1.3
NF-KB	−2.0	0.5	−2.9
P53	−7.5	0.0	2.4
HIF1A	−3.4	1.7	2.5
ERCC3	−2.9	−3.0	3.7
Skeletogenesis			
SM3O	0.0	0.0	−0.9
SM50	−4.5	0.3	−0.1
BMP5/7	−2.0	1.4	1.0
Nec	−2.7	2.2	1.2
uni	−2.5	1.5	−1.8
p16	−2.2	0.4	0.3
p19	−3.0	0.7	0.4
Jun	−2.3	0.7	0.7
Development/Differentiation			
hat	−0.9	−1.3	−0.7
sox9	−3.5	−1.2	1.9
BP10	−2.1	1.1	1.4
Blimp	−2.6	−0.1	−6.5
Alix	−3.8	−3.8	−4.5
Wnt5	−2.7	0.0	−1.2
Wnt6	−2.8	−0.5	−2.5
Wnt8	1.2	0.6	2.0
δ-2-catenin	−2.3	1.8	−2.6
Nodal	−2.5	1.1	−4.6
tcf4	−5.2	−1.1	3.1
TCF7	−2.0	0.3	−1.8
FoxG	−3.2	−0.5	−1.2
FoxA	3.5	0.5	−1.2
Fox0	4.1	1.0	−3.0
GFI1	−2.5	0.7	−0.6

Onecut	−2.1	1.2	0.7
TAK1	−3.5	3.9	5.0
VEGF	−2.2	−0.9	−1.8
JNK	−3.0	2.0	−0.5
Detoxification			
MT	−2.8	0.5	0.2
MT4	−1.7	0.7	−1.7
MT5	−0.4	1.2	2.3
MT6	−3.8	2.1	2.0
MT7	−2.7	0.7	−0.8
MT8	−8.3	1.7	−1.8
MDRI	0.1	1.6	−6.0
CAT	−2.5	0.6	−4.9
